# Survival prediction for heart failure complicated by sepsis: based on machine learning methods

**DOI:** 10.3389/fmed.2024.1410702

**Published:** 2024-10-03

**Authors:** Qitian Zhang, Lizhen Xu, Weibin He, Xinqi Lai, Xiaohong Huang

**Affiliations:** ^1^Department of Cardiology, Zhangzhou Affiliated Hospital of Fujian Medical University, Zhangzhou, Fujian, China; ^2^Department of Endocrinology, Shengli Clinical Medical College of Fujian Medical University, Fujian Provincial Hospital, Fuzhou University Affiliated Provincial Hospital, Fuzhou, China

**Keywords:** heart failure, sepsis, eICU-CRD, MIMIC-IV, machine learning, hospital mortality

## Abstract

**Background:**

Heart failure is a cardiovascular disorder, while sepsis is a common non-cardiac cause of mortality. Patients with combined heart failure and sepsis have a significantly higher mortality rate and poor prognosis, making early identification of high-risk patients and appropriate allocation of medical resources critically important.

**Methods:**

We constructed a survival prediction model for patients with heart failure and sepsis using the eICU-CRD database and externally validated it using the MIMIC-IV database. Our primary outcome is the 28-day all-cause mortality rate. The Boruta method is used for initial feature selection, followed by feature ranking using the XGBoost algorithm. Four machine learning models were compared, including Logistic Regression (LR), eXtreme Gradient Boosting (XGBoost), Adaptive Boosting (AdaBoost), and Gaussian Naive Bayes (GNB). Model performance was assessed using metrics such as area under the curve (AUC), accuracy, sensitivity, and specificity, and the SHAP method was utilized to visualize feature importance and interpret model results. Additionally, we conducted external validation using the MIMIC-IV database.

**Results:**

We developed a survival prediction model for heart failure complicated by sepsis using data from 3891 patients in the eICU-CRD and validated it externally with 2928 patients from the MIMIC-IV database. The LR model outperformed all other machine learning algorithms with a validation set AUC of 0.746 (XGBoost: 0.726, AdaBoost: 0.744, GNB: 0.722), alongside accuracy (0.685), sensitivity (0.666), and specificity (0.712). The final model incorporates 10 features: age, ventilation, norepinephrine, white blood cell count, total bilirubin, temperature, phenylephrine, respiratory rate, neutrophil count, and systolic blood pressure. We employed the SHAP method to enhance the interpretability of the model based on the LR algorithm. Additionally, external validation was conducted using the MIMIC-IV database, with an external validation AUC of 0.699.

**Conclusion:**

Based on the LR algorithm, a model was constructed to effectively predict the 28-day all-cause mortality rate in patients with heart failure complicated by sepsis. Utilizing our model predictions, clinicians can promptly identify high-risk patients and receive guidance for clinical practice.

## 1 Introduction

The 2018 medical insurance data reveals that sepsis and heart failure, respectively, ranked first and second in 30-day readmission rates among patients (1). Sepsis is defined as a dysregulated host response to infection, leading to organ failure (2). In 2017, an estimated 48.9 million cases of sepsis were recorded globally, resulting in 11 million sepsis-related deaths, which accounted for 19.7% of all global deaths (3). The mortality rates of sepsis in intensive care units and hospitals are reported to be 25.8 and 35.3%, respectively (4), with annual losses exceeding $24 billion (5, 6). Heart failure is a cardiovascular disorder characterized by high incidence and mortality rates, representing an escalating global epidemic (7). Over 64 million individuals worldwide are afflicted with heart failure, severely compromising their quality of life (8). Chronic heart failure is the leading complication in septic patients, with two-thirds of critically ill cases having prior heart failure (9, 10). Heart failure patients may exhibit underlying circulatory dysfunction and impaired cardiac reserve, placing them at increased risk if they develop sepsis. Alon et al. discovered that heart failure patients admitted for sepsis had a higher mortality rate compared to those without heart failure (51% vs. 41%; *p* = 0.015) (11). Walker et al. studied the effect of sepsis on heart failure patient mortality and found it caused one-fourth of deaths (12). The high incidence and mortality rates stress the importance of early identification, assessment, and management of heart failure patients with sepsis.

Currently, there are no identified predictive models for survival in patients with heart failure complicated by sepsis. The Sequential Organ Failure Assessment (SOFA), Simplified Acute Physiology Score II (SAPS II), and Acute Physiology Score III (APS III) are frequently utilized assessment tools for predicting disease prognosis (13, 14). Despite their extensive utilization, they exhibit limitations such as the complexity of assessment, insufficient specificity, and potential suitability restricted to specific disease types or clinical contexts. The current research trend is to integrate novel biomarkers (15, 16) into established scoring systems or to revamp these systems (17) to improve their predictive accuracy for disease prognosis. In clinical practice, machine learning is widely applied for result prediction, diagnosis, medical image interpretation, disease risk assessment, and treatment planning (18, 19). Compared to traditional statistical methods, machine learning excels in handling complex data, exhibiting higher accuracy and efficiency (20). In the past, the application of machine learning was constrained by limited interpretability. However, with the emergence of techniques like SHAP, users can now professionally understand model predictions with greater clarity (21).

Our research aims to build survival prediction models using various machine learning algorithms to assess the overall in-hospital mortality rate among patients with heart failure complicated by sepsis. We utilize the eICU-CRD database to build machine learning models, selecting the one with optimal predictive performance. Subsequently, we conduct external validation using the MIMIC-IV database. Additionally, the SHAP method is used to explain model predictions and assess the importance of features. The objective of this study is to identify critically ill patients and offer guidance for clinical practice.

## 2 Materials and methods

### 2.1 Data sources and study population

This study draws data from two primary sources: the eICU Collaborative Research Database (eICU-CRD) and The Medical Information Mart for Intensive Care IV database (MIMIC-IV). The eICU-CRD database encompasses various ICU units across the United States, offering a comprehensive array of clinical data, physiological parameters, and medical events. Spanning from 2014 to 2015, it meticulously documents information for over 200,000 patients, facilitating medical research endeavors and data-informed clinical decision-making (22). On the other hand, MIMIC-IV (version 2.2) represents an extensive repository of intensive care data, featuring detailed records of more than 190,000 ICU patients from 2008 to 2019 (23). This database is characterized by its exhaustive collection of clinical details, including demographic profiles, laboratory findings, and medication histories, serving as invaluable resources for rigorous clinical investigations.

We identified patients with heart failure complicated by sepsis from both the eICU-CRD and MIMIC-IV databases using ICD-9 and ICD-10 codes. The exclusion criteria for the study population are: (1) age under 18 years, (2) ICU stay duration less than 24 h, and (3) clinical information missing rate exceeding 30% at data collection. For patients with multiple hospital admissions or ICU visits, only the first ICU experience during the initial hospital admission is considered. Heart failure was defined as a syndrome resulting from structural or functional cardiac abnormalities that lead to inadequate cardiac output and congestion in the systemic or pulmonary circulation, encompassing all types of heart failure with different ejection fractions. Sepsis was diagnosed based on the Sepsis-3.0 guidelines, which define it as life-threatening organ dysfunction caused by a dysregulated host response to infection. A SOFA score ≥ 2 (or a qSOFA score ≥ 2 for suspected infection in non-ICU settings) was used to diagnose sepsis.

### 2.2 Data extraction and preprocessing

In this study, we included ICU patients diagnosed with heart failure and sepsis, and extracted the following data: (1) Demographics: age, gender, height, and weight; (2) Vital Signs: temperature (T), heart rate (HR), respiratory rate (R), systolic blood pressure (SBP), diastolic blood pressure (DBP), mean blood pressure (MBP), and peripheral oxygen saturation (SpO2); (3) Laboratory parameters: complete blood count, liver and kidney function tests, electrolytes, lipid profile, blood gas analysis, coagulation function, cardiac enzymes, and BNP; (4) Comorbidities: hypertension, diabetes mellitus, hyperlipidemia, chronic obstructive pulmonary disease (COPD), pneumonia, chronic kidney disease (CKD), and atrial fibrillation (AF); (5) Medication data: angiotensin-converting enzyme inhibitors/angiotensin II receptor blockers (ACEI/ARB), beta Blockers, furosemide, spironolactone, dobutamine, dopamine, epinephrine, milrinone, norepinephrine, and phenylephrine; and (6) Other Indicators: Ventilation and 24-h fluid balance. The primary outcome is the 28-day all-cause mortality rate.

Initially, we transformed certain indicators, such as computing BMI from height and weight and determining 28-day in-hospital mortality using hospitalization duration and survival status. Variables with over 30% missing data were removed, and missing values in the remaining features were imputed using KNN. Outliers were identified using the 1.5 times interquartile range method, particularly focusing on BMI, mechanical ventilation time, and 24-h fluid balance, and were subsequently removed. Additionally, Spearman correlation coefficients were calculated to evaluate variable relationships, while VIF values assessed multicollinearity. Variables with high correlation or VIF exceeding 5 underwent pre-screening. Continuous variables were standardized for model stability, and categorical variables were transformed into dummy variables via one-hot encoding. Despite minor sample imbalances in the outcome variable, we chose not to employ sample balancing techniques.

### 2.3 Model construction and evaluation

The Boruta method is used for initial feature screening, determining feature importance by comparing them with randomly generated “shadow features” (24). The XGBoost method is employed for importance ranking of the preliminarily selected features. Model construction and validation are conducted using the EICU dataset, with 10-fold cross-validation to generate training and validation sets, and Logistic Regression (LR), eXtreme Gradient Boosting (XGBoost), Adaptive Boosting (AdaBoost), and Gaussian Naive Bayes (GNB) models are established and validated. Model performance is evaluated on the validation set using metrics such as the area under the curve (AUC) for discrimination, calibration curve for accuracy, and DCA curve for clinical utility, as well as metrics including accuracy, sensitivity, specificity, positive predictive value, negative predictive value, and F1 score. The final predictive model is optimized using hyperparameter tuning and grid search. Additionally, the MIMIC dataset is utilized as external validation data, following the same data processing methods as the EICU dataset, with evaluation based on metrics including AUC, accuracy, sensitivity, and specificity to assess model generalization performance.

### 2.4 Model interpretation

SHAP (SHapley Additive exPlanations) is a technique based on game theory’s Shapley values (25). It’s used to interpret machine learning predictions by dissecting the contribution of each feature. This enhances model transparency and ensures fair decision-making. We employ SHAP to analyze the outcomes of our top-performing machine learning model. This method not only identifies crucial features for optimizing model performance but also provides detailed insights through feature contribution charts, summary plots, and explanations for individual predictions. These tools help us understand the extent of each feature’s influence, whether it’s positive or negative, and how they collectively impact model outcomes.

### 2.5 Statistical analysis

For continuous variables, we display using mean and standard deviation, and comparison is done using *t*-tests (or Wilcoxon rank-sum tests); for categorical variables, presentation is in percentages, and comparison is conducted using chi-square tests (or Fisher’s exact tests). A *p*-value <0.05 is deemed statistically significant. All statistical analyses were performed using R version 4.2.3 and Python version 3.11.4.

## 3 Results

### 3.1 Baseline characteristics

**Figure 1:**
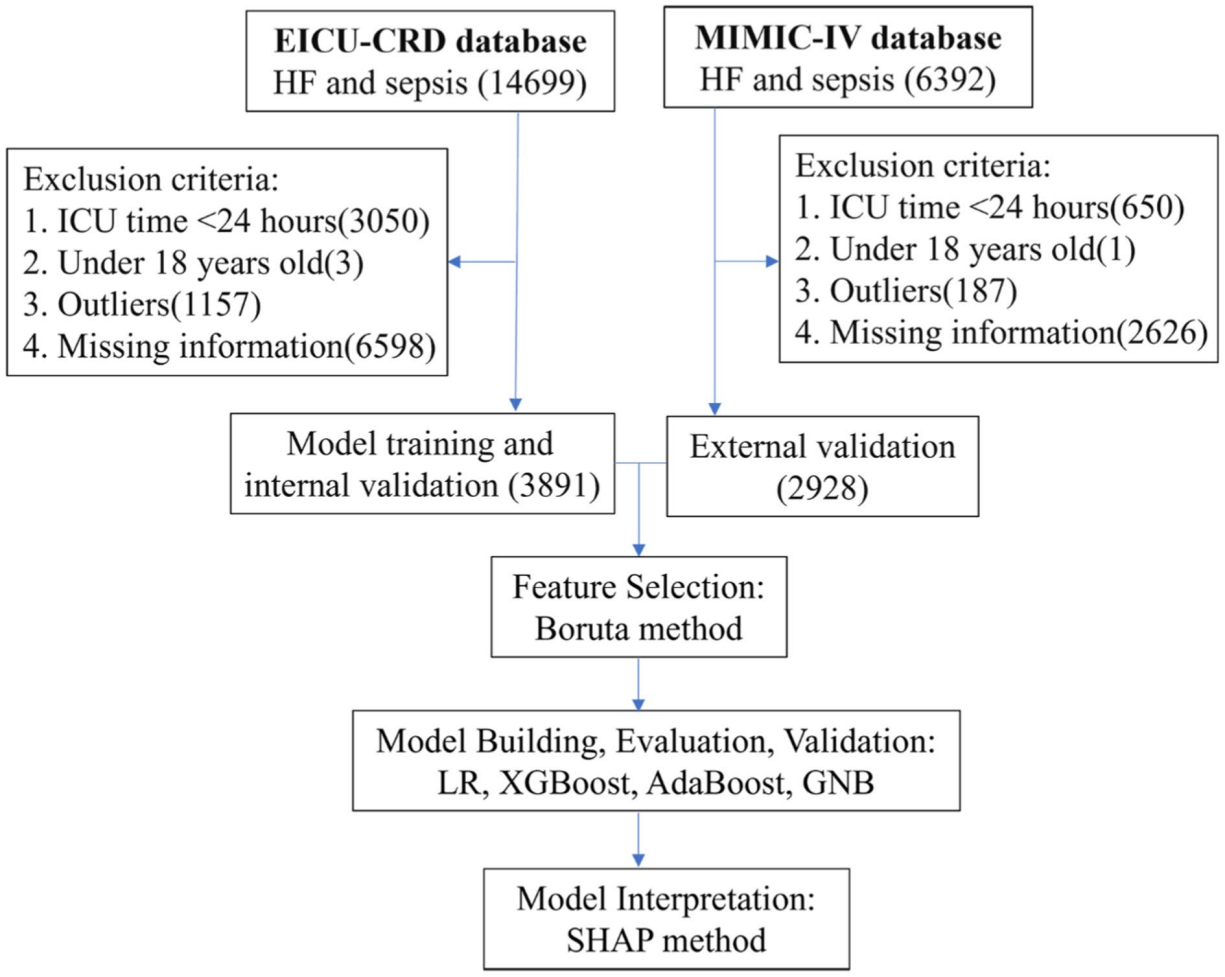
Flowchart of patient selection and research methodology.

According to the inclusion and exclusion criteria, our study cohort comprised a total of 6819 patients with heart failure and sepsis. Among these, 3891 cases from the eICU-CRD were used for model construction, while 2928 cases from the MIMIC-IV database were used for external validation. As shown in Figure 1, the screening process is illustrated. During the selection process, patients with ICU stays less than 1 day or under 18 years old were excluded. Subsequently, data processing involved removing outliers and handling missing values. In the eICU-CRD database, 560 patients (14.4%) died within 28 days, compared to 660 patients (22.5%) in the MIMIC-IV database. Differences in baseline characteristics are summarized in Table 1. In the eICU-CRD database, compared to the survival group, patients in the death group exhibited higher age, white blood cell count, neutrophil count, TBIL (total bilirubin), ALT (alanine aminotransferase), BUN (blood urea nitrogen), respiratory rate, fluid balance, and mechanical ventilation time, and lower BMI (body mass index), calcium, blood pressure, and peripheral oxygen pressure. Differences in comorbidities, such as atrial fibrillation, hypertension, and pneumonia, were also observed between the two groups. Additionally, there were differences in medication usage between the two groups, including the use of ACEI/ARB (ACE inhibitors/angiotensin receptor blockers), beta-blockers, furosemide, spironolactone, dobutamine, dopamine, epinephrine, norepinephrine, and phenylephrine.

**Table 1:** Baseline characteristics of the eICU-CRD and MIMIC-IV databases, categorized by survival and death groups.

	eICU-CRD			MIMIC-IV	
	Survival	Death	*p*	Survival	Death
	(*N* = 3331)	(*N* = 560)		(*N* = 2268)	(*N* = 660)
Age	69.5 (13.9)	74.5 (12.2)	<0.001	71.3 (13.4)	76.7 (11.2)
Gender			0.737		
0	1516 (45.5%)	250 (44.6%)		929 (41.0%)	283 (42.9%)
1	1815 (54.5%)	310 (55.4%)		1339 (59.0%)	377 (57.1%)
BMI	29.9 (7.63)	28.5 (7.12)	<0.001	29.2 (6.32)	28.0 (6.16)
WBC	11.5 (6.79)	15.5 (20.7)	<0.001	13.6 (8.91)	15.2 (10.3)
RBC	3.73 (0.77)	3.69 (0.76)	0.263	3.44 (0.77)	3.54 (0.79)
NE	77.7 (12.5)	81.1 (12.3)	<0.001	79.2 (10.6)	81.6 (12.0)
LYM	11.6 (8.84)	8.65 (8.24)	<0.001	12.1 (8.76)	8.44 (7.73)
MONO	7.36 (3.94)	6.70 (4.20)	0.001	5.03 (3.27)	5.35 (3.24)
PLT	202 (88.5)	200 (104)	0.649	196 (99.9)	212 (121)
Hb	10.9 (2.28)	10.9 (2.21)	0.941	10.2 (2.26)	10.4 (2.26)
Na	138 (5.27)	138 (6.19)	0.047	139 (4.94)	138 (6.21)
K	4.23 (0.78)	4.29 (0.81)	0.077	4.29 (0.73)	4.47 (0.88)
Cl	102 (6.68)	102 (7.60)	0.122	104 (6.66)	102 (7.61)
Ca	8.49 (0.74)	8.33 (0.89)	<0.001	8.29 (0.79)	8.25 (0.94)
GLU	151 (76.1)	155 (88.4)	0.341	151 (74.3)	169 (86.4)
TBIL	0.93 (0.91)	1.21 (1.35)	<0.001	1.01 (1.55)	1.57 (3.70)
ALT	90.1 (403)	179 (480)	<0.001	100 (466)	160 (509)
AST	124 (644)	266 (783)	<0.001	155 (698)	279 (1258)
BUN	36.0 (23.6)	42.3 (26.0)	<0.001	32.9 (25.1)	43.3 (28.6)
Cr	2.11 (2.09)	2.07 (1.49)	0.58	1.69 (1.63)	2.09 (1.75)
T	36.7 (0.79)	36.5 (1.02)	<0.001	36.7 (2.04)	36.5 (2.42)
HR	89.3 (21.0)	90.6 (21.9)	0.169	88.2 (19.6)	91.2 (21.2)
R	21.7 (6.71)	22.8 (7.09)	0.001	19.0 (6.42)	20.9 (6.55)
SBP	125 (28.4)	115 (26.2)	<0.001	118 (24.0)	116 (25.1)
DBP	69.4 (18.5)	64.9 (17.7)	<0.001	67.7 (144)	65.6 (19.2)
MBP	87.7 (19.7)	81.5 (18.3)	<0.001	77.5 (18.0)	79.4 (38.5)
SPO2	96.6 (4.80)	95.5 (7.82)	0.001	97.3 (18.7)	95.8 (5.93)
AF			<0.001		
0	2585 (77.6%)	382 (68.2%)		550 (24.3%)	162 (24.5%)
1	746 (22.4%)	178 (31.8%)		1718 (75.7%)	498 (75.5%)
CKD			0.137		
0	2749 (82.5%)	447 (79.8%)		1355 (59.7%)	385 (58.3%)
1	582 (17.5%)	113 (20.2%)		913 (40.3%)	275 (41.7%)
COPD			0.468		
0	2726 (81.8%)	466 (83.2%)		1121 (49.4%)	368 (55.8%)
1	605 (18.2%)	94 (16.8%)		1147 (50.6%)	292 (44.2%)
Diabetes			0.689		
0	3253 (97.7%)	549 (98.0%)		2061 (90.9%)	577 (87.4%)
1	78 (2.34%)	11 (1.96%)		207 (9.13%)	83 (12.6%)
Hyperlipidemia			0.687		
0	3104 (93.2%)	525 (93.8%)		1670 (73.6%)	415 (62.9%)
1	227 (6.81%)	35 (6.25%)		598 (26.4%)	245 (37.1%)
Hypertension			0.002		
0	2435 (73.1%)	445 (79.5%)		1464 (64.6%)	367 (55.6%)
1	896 (26.9%)	115 (20.5%)		804 (35.4%)	293 (44.4%)
Pneumonia			<0.001		
0	2676 (80.3%)	396 (70.7%)		1055 (46.5%)	287 (43.5%)
1	655 (19.7%)	164 (29.3%)		1213 (53.5%)	373 (56.5%)
ACEI/ARB			<0.001		
0	2839 (85.2%)	537 (95.9%)		1393 (61.4%)	574 (87.0%)
1	492 (14.8%)	23 (4.11%)		875 (38.6%)	86 (13.0%)
Betablockers			<0.001		
0	2132 (64.0%)	409 (73.0%)		341 (15.0%)	236 (35.8%)
1	1199 (36.0%)	151 (27.0%)		1927 (85.0%)	424 (64.2%)
Furosemide			<0.001		
0	1782 (53.5%)	346 (61.8%)		252 (11.1%)	142 (21.5%)
1	1549 (46.5%)	214 (38.2%)		2016 (88.9%)	518 (78.5%)
Spironolactone			0.001		
0	3177 (95.4%)	552 (98.6%)		2082 (91.8%)	624 (94.5%)
1	154 (4.62%)	8 (1.43%)		186 (8.20%)	36 (5.45%)
Dobutamine			<0.001		
0	3180 (95.5%)	505 (90.2%)		2100 (92.6%)	559 (84.7%)
1	151 (4.53%)	55 (9.82%)		168 (7.41%)	101 (15.3%)
Dopamine			0.001		
0	3186 (95.6%)	517 (92.3%)		2128 (93.8%)	554 (83.9%)
1	145 (4.35%)	43 (7.68%)		140 (6.17%)	106 (16.1%)
Epinephrine			<0.001		
0	3240 (97.3%)	520 (92.9%)		1814 (80.0%)	559 (84.7%)
1	91 (2.73%)	40 (7.14%)		454 (20.0%)	101 (15.3%)
Milrinone			0.86		
0	3232 (97.0%)	542 (96.8%)		1351 (59.6%)	214 (32.4%)
1	99 (2.97%)	18 (3.21%)		917 (40.4%)	446 (67.6%)
Norepinephrine			<0.001		
0	2871 (86.2%)	362 (64.6%)		1344 (59.3%)	370 (56.1%)
1	460 (13.8%)	198 (35.4%)		924 (40.7%)	290 (43.9%)
Phenylephrine			<0.001		
0	3215 (96.5%)	495 (88.4%)		2024 (89.2%)	612 (92.7%)
1	116 (3.48%)	65 (11.6%)		244 (10.8%)	48 (7.27%)
Ventilation			<0.001	111 (145)	119 (110)
0	2338 (70.2%)	272 (48.6%)			
1	993 (29.8%)	288 (51.4%)		88 (3.88%)	50 (7.58%)
Ventilation hour	357 (1664)	819 (2425)	<0.001	2180 (96.1%)	610 (92.4%)
Balance	−520.14 (4968)	1272 (7270)	<0.001	1991 (4988)	2771 (3971)

BMI, Body mass index; WBC, White blood cell; RBC, Red blood cell; NE, Neutrophil; LYM, Lymphocyte; MONO, Monocyte; PLT, Platelet; Hb, Hemoglobin; Na, Sodium; K, Potassium; Cl, Chloride; Ca, Calcium; GLU, Glucose; TBIL, Total bilirubin; ALT, Alanine aminotransferase; AST, Aspartate aminotransferase; BUN, Blood urea nitrogen; Cr, Creatinine; T, Temperature; HR, Heart rate; R, Respiratory rate; SBP, Systolic blood pressure; DBP, Diastolic blood pressure; MBP, Mean blood pressure; SPO2, Oxygen saturation; AF, Atrial fibrillation; CKD, Chronic kidney disease; COPD, Chronic obstructive pulmonary disease; ACEI/ARB, Angiotensin-converting enzyme inhibitor/angiotensin receptor blocker; betablockers, Beta-blockers; balance: fluid balance in the previous 24 h.

### 3.2 Feature selection

We eliminated features with a missing rate exceeding 30%, as demonstrated in Appendix Figure 1. Features with notably high missing rates are primarily found in laboratory tests such as cardiac enzymes, blood gas analysis, lipid profile, and coagulation function. Additionally, guided by the correlation heatmap showing features with correlation coefficients greater than 0.5 and features with VIF exceeding 5, as illustrated in Appendix Figure 2, we conducted further screening. Prior to model construction, we excluded features with high correlation and VIF, including hemoglobin, lymphocyte count, chloride, aspartate aminotransferase, blood urea nitrogen, mean blood pressure, and diastolic blood pressure.

**Figure 2:**
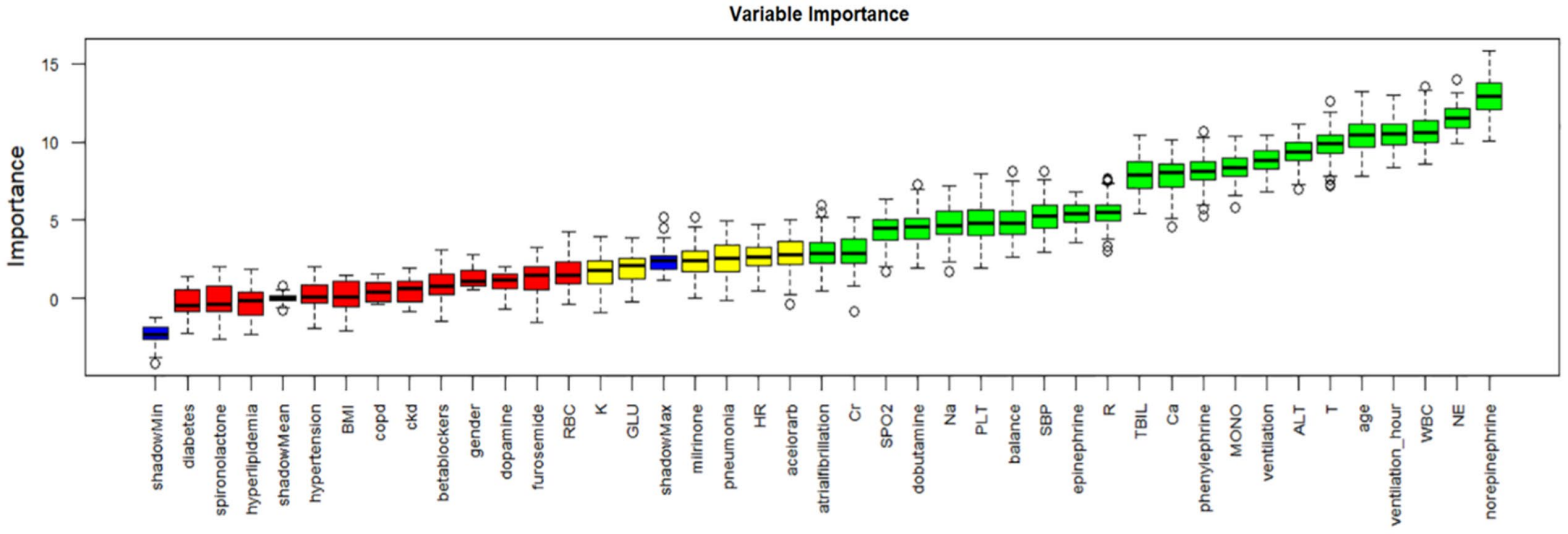
Feature selection analyzed by Boruta algorithm.

The Boruta method, based on random forests, assesses feature importance by comparing original features with randomly generated “shadow features.” We applied Boruta for initial feature selection, as depicted in Figure 2. Green denotes important features included in the model to enhance predictive capability; red represents unimportant features excluded from consideration; yellow indicates features with uncertain importance requiring further investigation. Blue represents shadow features for comparison but not used in model training. Boruta identified 22 initial features, including age, WBC (white blood cell count), NE (neutrophil count), MONO (monocyte count), PLT (platelet count), sodium, calcium, TBIL (total bilirubin), alanine aminotransferase, creatinine, T (temperature), R (respiratory rate), SBP (systolic blood pressure), oxygen saturation, ventilation time, BMI, atrial fibrillation, dopamine, epinephrine, norepinephrine, phenylephrine, and ventilation.

The XGBoost algorithm ranks feature importance based on split frequency and gain in decision trees. Appendix Figure 3 shows our feature importance ranking using XGBoost. The top 10 variables, in descending order of importance, are: age, ventilation, norepinephrine, WBC, TBIL, T, phenylephrine, R, NE, SBP.

### 3.3 Model construction

**Figure 3:**
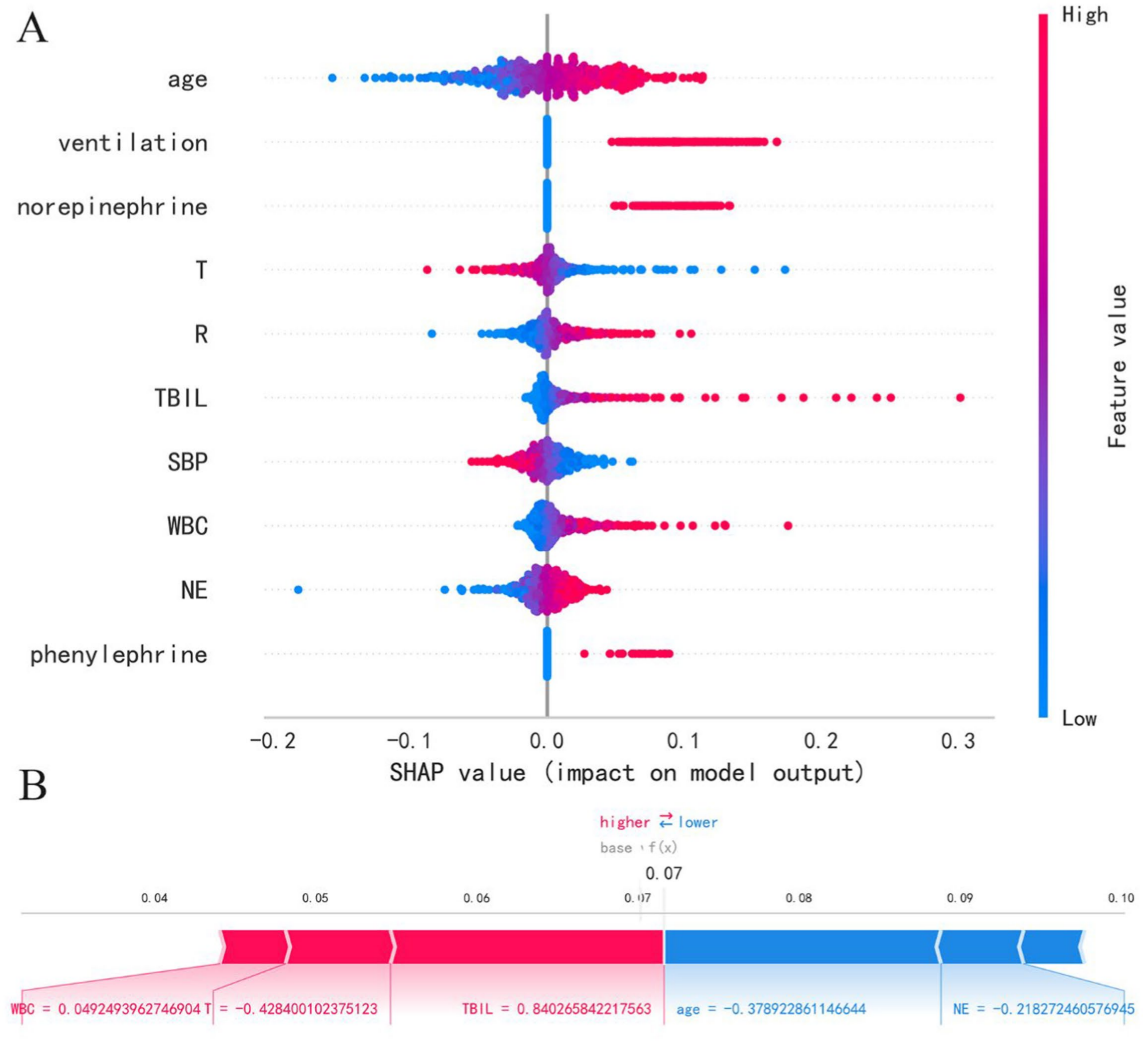
**(A)** SHAP summary plot and **(B)** SHAP force plot.

**Figure 4:**
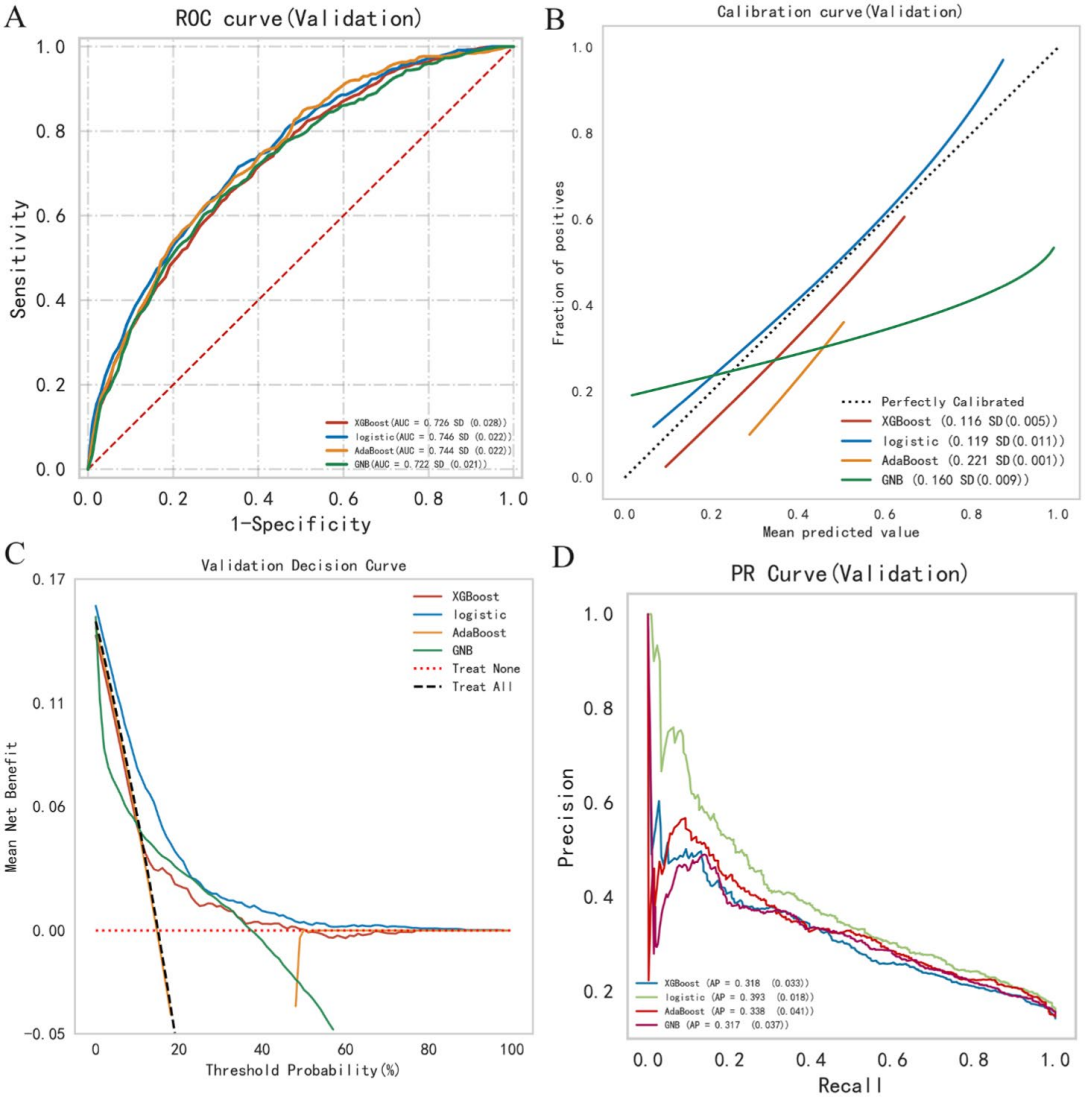
Summary plot of machine learning performance evaluation. **(A)** ROC curve, **(B)** calibration plot, **(C)** DCA curve, **(D)** PR curve.

This study utilized four binary classification machine learning algorithms, Logistic Regression (LR), eXtreme Gradient Boosting (XGBoost), Adaptive Boosting (AdaBoost), and Gaussian Naive Bayes (GNB), to construct predictive models. Employing the eICU database, we implemented a 10-fold cross-validation technique to establish training and validation sets, followed by evaluation on a separate test cohort. Figure 3 and Table 2 illustrate the performance of these models. The ROC curve (Figure 4A) highlights LR’s superior performance, achieving an AUC of 0.746 in the test cohort, compared to XGBoost (0.726), AdaBoost (0.744), and GNB (0.722). Furthermore, LR’s calibration curve (Figure 4B) closely aligns with the ideal line, indicating excellent calibration. Decision curve analysis (DCA) (Figure 4C) indicates LR’s highest net benefit within the 0–80% threshold range. The precision-recall (PR) curve (Figure 4D) illustrates LR’s higher recall at sustained high precision. Additionally, LR demonstrates robust performance across various metrics, including accuracy (0.685), sensitivity (0.666), specificity (0.712), positive predictive value (0.285), negative predictive value (0.914), and F1 score (0.397). Consequently, we selected the LR algorithm for model construction, incorporating 10 variables: age, ventilation, norepinephrine, WBC, TBIL, T, phenylephrine, R, NE, and SBP. Through hyperparameter tuning and grid search optimization, we established the model parameters as follows: tol (convergence measure): 1e-06, penalty (regularization type): l2, max_iter (number of iterations): 100, C (regularization factor): 1.0.

**Table 2:** Model performance comparison: AUC, accuracy, sensitivity, specificity, PPV, NPV, F1 score, and Brier score.

Models	AUC	Accuracy	Sensitivity	Specificity	PPV	NPV	F1 Score	Brierscore
Validation set								
LR	0.746	0.685	0.666	0.712	0.285	0.914	0.397	0.119
XGBoost	0.726	0.718	0.710	0.637	0.268	0.910	0.388	0.116
AdaBoost	0.744	0.672	0.724	0.645	0.265	0.922	0.387	0.221
GNB	0.722	0.683	0.644	0.702	0.269	0.914	0.377	0.160
External validation								
LR	0.699	0.403	0.673	0.648	0.261	0.896	0.376	0.169

LR, Logistic regression; XGBoost, eXtreme gradient boosting; AdaBoost, Adaptive boosting; GNB, Gaussian Naive Bayes; AUC, Area under the curve; PPV, Positive predictive value; NPV, Negative predictive value.

### 3.4 Model interpretation

This study employs the SHAP method to interpret model results, presenting both SHAP summary plots and SHAP force plots. In the SHAP summary plot, the Y-axis represents features, while the X-axis indicates the impact of features on outcomes. Each point represents a sample, with red indicating high-risk values and blue indicating low-risk values. As shown in Figure 3A, the LR model’s feature importance from top to bottom is: age, ventilation, norepinephrine, T, R, TBIL, SBP, WBC, NE, phenylephrine. Older age (red points) correlates with higher SHAP estimated values, predicting an increased risk of mortality. Additionally, higher white blood cell count, total bilirubin, and respiratory rate are associated with increased mortality risk. Patients using ventilation, norepinephrine, and phenylephrine also show increased mortality risk. Furthermore, lower temperature and lower systolic blood pressure are associated with increased mortality risk. In the SHAP force plot, each Shapley value is represented by an arrow, indicating whether it positively (increases) or negatively (decreases) affects the prediction. As illustrated in Figure 3B, increases in white blood cell count, decreases in temperature, and increases in total bilirubin push the predicted mortality risk higher, while younger age and lower neutrophil count push the predicted mortality risk lower. It’s important to note that due to standardization of numerical variables to a mean of 0 and a variance of 1, the data in the plots are not in their original scale.

### 3.5 External validation

We selected 2928 cases of patients with heart failure and sepsis from the MIMIC-IV database for external validation. In the MIMIC database, these patients had a 28-day in-hospital mortality rate of 22.5%, slightly lower than that of the eICU-CRD database (14.4%). Prior to external validation, we applied the same data processing methods to the MIMIC data as we did to the eICU-CRD data. The validation results revealed an AUC of 0.699 and a Brier score of 0.169. Additionally, the accuracy, sensitivity, specificity, positive predictive value, negative predictive value, and F1 score were 0.699, 0.156, 0.403, 0.673, 0.648, and 0.261, respectively. With the AUC difference between the external validation and validation/test sets being less than 0.1, we conclude that the LR model demonstrates favorable stability.

## 4 Discussion

This study represents the pioneering application of machine learning algorithms to forecast in-hospital mortality among patients with heart failure and sepsis. Our model can be applied to heart failure patients with sepsis upon ICU admission. Our model exhibits exceptional performance in distinguishing between survival and mortality outcomes, coupled with robust calibration and clinical relevance. The utilization of external validation bolsters the model’s reliability and generalizability, validating its efficacy across diverse datasets and fortifying the study’s scientific robustness and credibility. Leveraging SHAP for visual interpretation of model outcomes enhances the interpretability of predictive results. Furthermore, the model’s reliance on a concise and readily accessible set of predictive variables underscores its suitability for clinical deployment. Our model could be integrated into clinical decision support systems within hospitals, especially in ICU. The model would automatically calculate the mortality risk for patients with heart failure complicated by sepsis using routinely collected clinical data, with outputs presented to clinicians via the electronic health record system. This would provide real-time risk assessments to help prioritize care and optimize resource allocation.

Our research findings suggest that the Logistic Regression (LR) model exhibits superior performance in predicting the survival rate of patients with heart failure complicated by sepsis. Moreover, studies indicate that the LR algorithm performs effectively in forecasting various clinical binary outcomes (26, 27). LR offers several advantages, including its simplicity, broad applicability, and straightforward result interpretation, establishing it as a pivotal and dependable modeling technique for binary classification problems (28). However, LR has its limitations; it is sensitive to the quality of feature engineering, vulnerable to outliers, and unable to handle complex non-linear relationships. Moreover, in situations with large feature spaces and predominantly sparse features, LR’s performance may be limited, potentially resulting in overfitting (29). Before model construction, we conducted comprehensive data preprocessing, encompassing correlation and multicollinearity assessments, outlier and missing value handling, and data standardization, aiming to enhance the LR model’s efficacy. Additionally, external validation has confirmed that the LR model we constructed avoids overfitting and demonstrates reliable generalization ability.

Sepsis and heart failure are common complications in critically ill patients, characterized by complex pathological conditions. Cardiac dysfunction in sepsis, indicated by reduced EF, may accelerate the progression to septic shock by lowering cardiac output and metabolic demand (30). Treatment strategies for sepsis and heart failure often conflict, influenced by varying severity and patient conditions (31). Fluid resuscitation, recommended in sepsis management guidelines, addresses tissue perfusion deficits but may exacerbate congestive symptoms and worsen prognosis in heart failure (32, 33). Our study indicates that higher fluid balance predicts increased mortality in heart failure and sepsis. Singh et al. found that septic patients receiving >3L of fluid experienced reduced EF and higher in-hospital mortality (34). Additionally, other studies have shown that higher fluid balance during hospitalization is associated with increased mortality in patients with heart failure combined with sepsis (35–37). Zhang et al. discovered that higher fluid balance within 24 h of admission is strongly associated with in-hospital mortality in patients with heart failure and sepsis (OR 2.53, 95% CI 1.60–3.99, *p* < 0.001) (31). Due to myocardial edema and oxidative stress, excessive fluid intake is a factor contributing to myocardial injury. For patients with high fluid balance, increased atrial and venous pressures can lead to fluid shift into the interstitium, exacerbating tissue edema, causing tissue distortion and microcirculatory disturbances, thereby resulting in cellular metabolic dysregulation (38, 39). There remains controversy surrounding fluid resuscitation. Duttuluri et al. retrospectively evaluated heart failure patients with severe sepsis, finding increased in-hospital mortality and intubation rates in the hypotensive subgroup receiving inadequate fluid (<30 mL/kg) (40).

Our research reveals that patients with elevated respiratory rates, hypotension, and those necessitating interventions such as norepinephrine, phenylephrine, or ventilation, exhibit a heightened risk of mortality prediction. Norepinephrine and phenylephrine are typically employed to augment cardiac contractility and blood pressure for organ perfusion maintenance, while ventilation is essential for respiratory support. This elevated predictive risk likely reflects the severity of patients’ conditions and the associated potential hazards they face. Moreover, it underscores the necessity for prompt and assertive therapeutic interventions tailored to these patients, alongside vigilant monitoring and comprehensive support measures. Sepsis guidelines recommend norepinephrine as the first-line vasopressor for sepsis and septic shock (33). De Backer et al. found in their study that among 280 cases of cardiogenic shock patients, norepinephrine was more effective than dopamine, significantly reducing the 28-day mortality rate (*p* = 0.03) (41). Additionally, a meta-analysis from 2015 also indicated that in the treatment of septic shock, norepinephrine, compared to dopamine, could lower the mortality rate (RR: 0.89; 95% CI: 0.81–0.98) (42). Additionally, there are studies indicating that compared to adrenaline, norepinephrine carries a lower risk of tachycardia [29] and is associated with reduced mortality risk (43, 44).

With the exacerbation of an aging society, the incidence of sepsis among the elderly is gradually increasing, making it one of the leading causes of mortality in this demographic (45). Age has been demonstrated as an independent risk factor for mortality in sepsis patients, with mortality rates showing a linear increase with advancing age (46). Our research findings indicate that advanced age is associated with a higher predictive risk of mortality in patients with sepsis complicated by heart failure. Elderly patients commonly exhibit compromised immune function, diminished organ reserve, and a higher prevalence of comorbidities such as diabetes and coronary artery disease compared to younger counterparts (47). Sepsis in this demographic frequently presents and swiftly evolves into multi-organ failure. De Matteis et al. studied 6930 elderly patients with heart failure and found that in-hospital mortality increased with advancing age, with infection correlating with an elevated risk of in-hospital death (48). We also found that elevated levels of white blood cells and norepinephrine were associated with poor outcomes. In bacterial and fungal infections, elevated blood neutrophil levels serve as early and sensitive indicators of inflammation (49). Elevated white blood cell or neutrophil counts in sepsis patients suggest immune system activation and intensified inflammatory response, potentially indicating an excessively activated inflammatory state associated with increased mortality risk. Heightened vigilance and proactive therapeutic interventions are warranted to mitigate inflammation and prevent further deterioration in such cases. Additionally, low body temperature and elevated total bilirubin increase the risk of mortality assessment. Observing low body temperature or elevated total bilirubin (TBIL) in sepsis patients may suggest a severe condition and poor prognosis. Low body temperature could indicate suppressed inflammatory response or impaired metabolic function, compromising the body’s resistance to infection. Elevated TBIL may signify impaired liver function, possibly due to infection or inflammation.

This study has several limitations. Firstly, we acknowledge that the quality and completeness of the data in the MIMIC-IV and eICU-CRD databases may have certain limitations, especially with the potential absence of key clinical variables (such as ejection fraction or NT-proBNP), which could affect the accuracy of the model’s predictions. Future studies will need to incorporate more comprehensive data to improve the model and conduct further validation to enhance its accuracy. Secondly, as this is a retrospective study, the data primarily comes from ICU patients, which may introduce selection bias, limiting the model’s broader applicability to other clinical settings. Therefore, we suggest that future research validate the model using multi-center data to reduce selection bias and improve its generalizability. Additionally, the imbalance between survival and death in the dataset may affect the model’s performance in predicting mortality. Lastly, the data were collected at different time points, leading to potential temporal discrepancies, which may cause data drift and result in inconsistent model performance across different periods. Thus, future research should validate and adjust the model using data from various timeframes to address these challenges and ensure the model’s robustness in different temporal and clinical settings.

## 5 Conclusion

In this study, we constructed a machine learning model to predict 28-day all-cause mortality in ICU patients with heart failure complicated by sepsis. The final Logistic Regression model incorporates commonly used clinical indicators such as age, mechanical ventilation, respiratory rate, blood pressure, white blood cell count, and vasopressor use. This combination of variables enables the model to predict short-term mortality risk early, upon ICU admission, providing a clinical alert for high-risk patients and assisting clinicians in more effectively allocating medical and nursing resources. Furthermore, the model’s generalizability and potential clinical utility were validated across two large ICU databases (eICU-CRD and MIMIC-IV). Despite its strong predictive performance, further updates and validations with larger, multicenter patient cohorts are required to enhance the model’s generalizability and practical application in broader clinical settings.

## Data Availability

The datasets presented in this study can be found in online repositories. The names of the repository/repositories and accession number(s) can be found in the article/Supplementary material.

## References

[1] Hajj J Blaine N Salavaci J Jacoby D. The "Centrality of Sepsis": A review on incidence, mortality, and cost of care. Healthcare. (2018) 6. doi: 10.3390/healthcare6030090, PMID: 30061497 PMC6164723

[2] Napolitano LM. Sepsis 2018: definitions and guideline changes. Surg Infect. (2018) 19:117–25. doi: 10.1089/sur.2017.278 29447109

[3] Rudd KE Johnson SC Agesa KM Shackelford KA Tsoi D Kievlan DR. Global, regional, and national sepsis incidence and mortality, 1990-2017: analysis for the global burden of disease study. Lancet. (2020) 395:200–11. doi: 10.1016/S0140-6736(19)32989-7, PMID: 31954465 PMC6970225

[4] Vincent JL Marshall JC Namendys-Silva SA Francois B Martin-Loeches I Lipman J. Assessment of the worldwide burden of critical illness: the intensive care over nations (ICON) audit. Lancet Respir Med. (2014) 2:380–6. doi: 10.1016/S2213-2600(14)70061-X, PMID: 24740011

[5] Liu V Escobar GJ Greene JD Soule J Whippy A Angus DC. Hospital deaths in patients with sepsis from 2 independent cohorts. JAMA. (2014) 312:90–2. doi: 10.1001/jama.2014.5804 24838355

[6] Liang L Moore B Soni A. National Inpatient Hospital Costs: the Most expensive conditions by payer, 2017 In: Healthcare cost and utilization project (HCUP) statistical briefs. Rockville, MD: Agency for Healthcare Research and Quality (US) (2006) 32833416

[7] Gupta AK Tomasoni D Sidhu K Metra M Ezekowitz JA. Evidence-based Management of Acute Heart Failure. Can J Cardiol. (2021) 37:621–31. doi: 10.1016/j.cjca.2021.01.002 33440229

[8] Groenewegen A Rutten FH Mosterd A Hoes AW. Epidemiology of heart failure. Eur J Heart Fail. (2020) 22:1342–56. doi: 10.1002/ejhf.1858, PMID: 32483830 PMC7540043

[9] Pandolfi F Guillemot D Watier L Brun-Buisson C. Trends in bacterial sepsis incidence and mortality in France between 2015 and 2019 based on National Health Data System (Systeme national des donnees de Sante (SNDS)): a retrospective observational study. BMJ Open. (2022) 12:e058205. doi: 10.1136/bmjopen-2021-058205, PMID: 35613798 PMC9125708

[10] Jentzer JC van Diepen S Hollenberg SM Lawler PR Kashani KB. Shock severity assessment in cardiac intensive care unit patients with Sepsis and mixed septic-cardiogenic shock. Mayo Clin Proc Innov Qual Outcomes. (2022) 6:37–44. doi: 10.1016/j.mayocpiqo.2021.11.008, PMID: 35005436 PMC8715298

[11] Alon D Stein GY Korenfeld R Fuchs S. Predictors and outcomes of infection-related hospital admissions of heart failure patients. PLoS One. (2013) 8:e72476. doi: 10.1371/journal.pone.0072476 24009684 PMC3751916

[12] Walker AMN Drozd M Hall M Patel PA Paton M Lowry J. Prevalence and predictors of Sepsis death in patients with chronic heart failure and reduced left ventricular ejection fraction. J Am Heart Assoc. (2018) 7:e009684. doi: 10.1161/JAHA.118.009684, PMID: 30371261 PMC6474963

[13] Kopczynska M Sharif B Cleaver S Spencer N Kurani A Lee C. Red-flag sepsis and SOFA identifies different patient population at risk of sepsis-related deaths on the general ward. Medicine. (2018) 97:e13238. doi: 10.1097/MD.0000000000013238, PMID: 30544383 PMC6310498

[14] Le Gall JR Lemeshow S Saulnier F. A new simplified acute physiology score (SAPS II) based on a European/north American multicenter study. JAMA. (1993) 270:2957–63. doi: 10.1001/jama.270.24.2957, PMID: 8254858

[15] Yu H Nie L Liu A Wu K Hsein YC Yen DW. Combining procalcitonin with the qSOFA and sepsis mortality prediction. Medicine. (2019) 98:e15981. doi: 10.1097/MD.0000000000015981 31169735 PMC6571275

[16] Olejarova M Dobisova A Suchankova M Tibenska E Szaboova K Koutun J. Vitamin D deficiency - a potential risk factor for sepsis development, correlation with inflammatory markers, SOFA score and higher early mortality risk in sepsis. Bratislavske Lekarske Listy. (2019) 120:284–90. doi: 10.4149/BLL_2019_040 31023051

[17] Hou N Li M He L Xie B Wang L Zhang R. Predicting 30-days mortality for MIMIC-III patients with sepsis-3: a machine learning approach using XGboost. J Transl Med. (2020) 18:462. doi: 10.1186/s12967-020-02620-5, PMID: 33287854 PMC7720497

[18] Ramesh AN Kambhampati C Monson JR Drew PJ. Artificial intelligence in medicine. Ann R Coll Surg Engl. (2004) 86:334–8. doi: 10.1308/147870804290, PMID: 15333167 PMC1964229

[19] Deo RC. Machine learning in medicine. Circulation. (2015) 132:1920–30. doi: 10.1161/CIRCULATIONAHA.115.001593, PMID: 26572668 PMC5831252

[20] Lee A Taylor P Kalpathy-Cramer J Tufail AJO. Machine learning has arrived! Ophthalmol Retina. (2017) 124:1726–8. doi: 10.1016/j.ophtha.2017.08.046 29157423

[21] Lubo-Robles D Devegowda D Jayaram V Bedle H Marfurt KJ Pranter MJ. (2020). Machine learning model interpretability using SHAP values: application to a seismic facies classification task. SEG Int Expos Ann Meet.

[22] Pollard TJ Johnson AEW Raffa JD Celi LA Mark RG Badawi O. The eICU collaborative research database, a freely available multi-center database for critical care research. Sci Data. (2018) 5:180178. doi: 10.1038/sdata.2018.178 30204154 PMC6132188

[23] Johnson A Bulgarelli L Pollard T Horng S Celi LA. Mark RJPAoahpocm. Mimic-iv. (2020):49–55.

[24] Degenhardt F Seifert S Szymczak S. Evaluation of variable selection methods for random forests and omics data sets. Brief Bioinform. (2019) 20:492–503. doi: 10.1093/bib/bbx124, PMID: 29045534 PMC6433899

[25] Lundberg SM Lee S-I. A unified approach to interpreting model predictions. arXiv. (2017) 30:7874. doi: 10.48550/arXiv.1705.07874

[26] Liu L. (2018). Research on logistic regression algorithm of breast cancer diagnose data by machine learning. 2018 international conference on Robots & Intelligent System (ICRIS).

[27] Nishadi AST. Predicting heart diseases in logistic regression of machine learning algorithms by Python Jupyterlab. Comput Sci Med. (2019) 3:1–6.

[28] Maalouf M. Logistic regression in data analysis: an overview. Int. J. Data Anal Tech Strat. (2011) 3:281–99. doi: 10.1504/IJDATS.2011.041335

[29] Bisong E. Building machine learning and deep learning models on Google cloud platform. Berlin: Springer (2019).

[30] Ferro TN Goslar PW Romanovsky AA Petersen SR. Smoking in trauma patients: the effects on the incidence of sepsis, respiratory failure, organ failure, and mortality. J Trauma. (2010) 69:308–12. doi: 10.1097/TA.0b013e3181e1761e, PMID: 20699738

[31] Zhang B Guo S Fu Z Wu N Liu Z. Association between fluid balance and mortality for heart failure and sepsis: a propensity score-matching analysis. Anesthesiology. (2022) 22:324. doi: 10.1186/s12871-022-01865-5, PMID: 36273128 PMC9587660

[32] Levy MM Evans LE Rhodes A. The surviving Sepsis campaign bundle: 2018 update. Eur J Intensive Care Med. (2018) 44:925–8. doi: 10.1007/s00134-018-5085-0, PMID: 29675566

[33] Rhodes A Evans LE Alhazzani W Levy MM Antonelli M Ferrer R. Surviving sepsis campaign: international guidelines for management of sepsis and septic shock: 2016. Intensive Care Med. (2017) 43:304–77. doi: 10.1007/s00134-017-4683-6 28101605

[34] Singh H Iskandir M Sachdev S Simmons B Rabines A Hassen GW. The effect of initial volume resuscitation for Sepsis in patients with congestive heart failure: is it associated with higher mortality. J Card Fail. (2016) 22:S54–5. doi: 10.1016/j.cardfail.2016.06.161

[35] Shen Y Huang X Zhang WJCC. Association between fluid intake and mortality in critically ill patients with negative fluid balance: a retrospective cohort study. Crit Care. (2017) 21:1–8. doi: 10.1186/s13054-017-1692-3 28494815 PMC5427534

[36] Boyd JH Forbes J Nakada TA Walley KR Russell JA. Fluid resuscitation in septic shock: a positive fluid balance and elevated central venous pressure are associated with increased mortality. Crit Care Med. (2011) 39:259–65. doi: 10.1097/CCM.0b013e3181feeb15, PMID: 20975548

[37] Micek ST McEvoy C McKenzie M Hampton N Doherty JA Kollef MH. Fluid balance and cardiac function in septic shock as predictors of hospital mortality. Crit Care. (2013) 17:R246–9. doi: 10.1186/cc13072, PMID: 24138869 PMC4056694

[38] Marik PE Byrne L van Haren F. Fluid resuscitation in sepsis: the great 30 mL per kg hoax. J Thorac Dis. (2020) 12:S37–47. doi: 10.21037/jtd.2019.12.84, PMID: 32148924 PMC7024756

[39] Prowle JR Kirwan CJ Bellomo R. Fluid management for the prevention and attenuation of acute kidney injury. Nat Rev Nephrol. (2014) 10:37–47. doi: 10.1038/nrneph.2013.232, PMID: 24217464

[40] Duttuluri M Rose K Shapiro J Mathew J Jean R Kurtz S. Fluid resuscitation dilemma in patients with congestive heart failure presenting with severe sepsis/septic shock. D45 critical care: Circulatory hemodymanics, shock, cardiovascular disease, and fluid management. New York, NY: American Thoracic Society (2016). A7048 p.

[41] De Backer D Biston P Devriendt J Madl C Chochrad D Aldecoa C. Comparison of dopamine and norepinephrine in the treatment of shock. N Engl J Med. (2010) 362:779–89. doi: 10.1056/NEJMoa0907118 20200382

[42] Avni T Lador A Lev S Leibovici L Paul M Grossman A. Vasopressors for the treatment of septic shock: systematic review and Meta-analysis. PLoS One. (2015) 10:e0129305. doi: 10.1371/journal.pone.0129305, PMID: 26237037 PMC4523170

[43] Léopold V Gayat E Pirracchio R Spinar J Parenica J Tarvasmäki T. Epinephrine and short-term survival in cardiogenic shock: an individual data meta-analysis of 2583 patients. Eur J Intensive Care Med. (2018) 44:847–56. doi: 10.1007/s00134-018-5222-9 29858926

[44] Léopold V Gayat E Pirracchio R Spinar J Parenica J Tarvasmäki T. Correction to: epinephrine and short-term survival in cardiogenic shock: an individual data meta-analysis of 2583 patients. Intensive Care Med. (2018) 44:2022–3. doi: 10.1007/s00134-018-5372-9, PMID: 30215188

[45] Rowe TA McKoy JM. Sepsis in Older Adults. Infect Dis Clin N Am. (2017) 31:731–42. doi: 10.1016/j.idc.2017.07.010 29079157

[46] Martin GS Mannino DM Moss M. The effect of age on the development and outcome of adult sepsis. Crit Care Med. (2006) 34:15–21. doi: 10.1097/01.ccm.0000194535.82812.ba 16374151

[47] Carbajal-Guerrero J Cayuela-Dominguez A Fernandez-Garcia E Aldabo-Pallas T Marquez-Vacaro JA Ortiz-Leyba C. Epidemiology and long-term outcome of sepsis in elderly patients. Med Intensiva. (2014) 38:21–32. doi: 10.1016/j.medin.2012.12.006, PMID: 23462427

[48] de Matteis G Covino M Burzo ML Della Polla DA Franceschi F Mebazaa A. Clinical characteristics and predictors of in-hospital mortality among older patients with acute heart failure. JCM. (2022) 11:439. doi: 10.3390/jcm11020439, PMID: 35054133 PMC8781633

[49] Honda T Uehara T Matsumoto G Arai S Sugano MJCCA. Neutrophil left shift and white blood cell count as markers of bacterial infection. Clin Chim Acta. (2016) 457:46–53. doi: 10.1016/j.cca.2016.03.017, PMID: 27034055

