# Supplementary figures and images for: Survival prediction for heart failure complicated by sepsis: based on machine learning methods
Source: Front Med (Lausanne). 2024 Oct 3;11:1410702. doi: 10.3389/fmed.2024.1410702 (PMC11484001; doi:10.3389/fmed.2024.1410702)

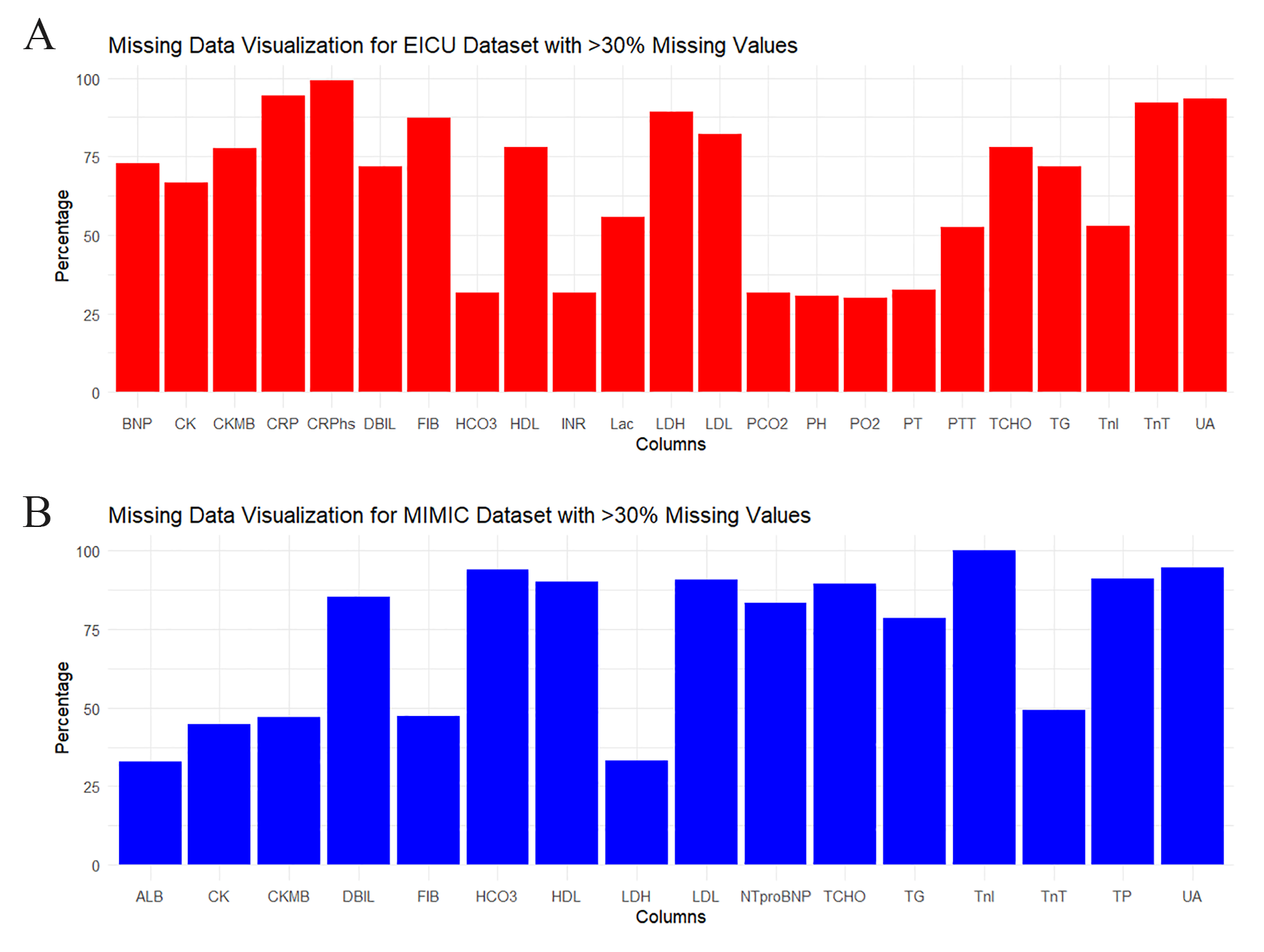

Supplement: Supplementary file 2 [file Image_1.TIF]

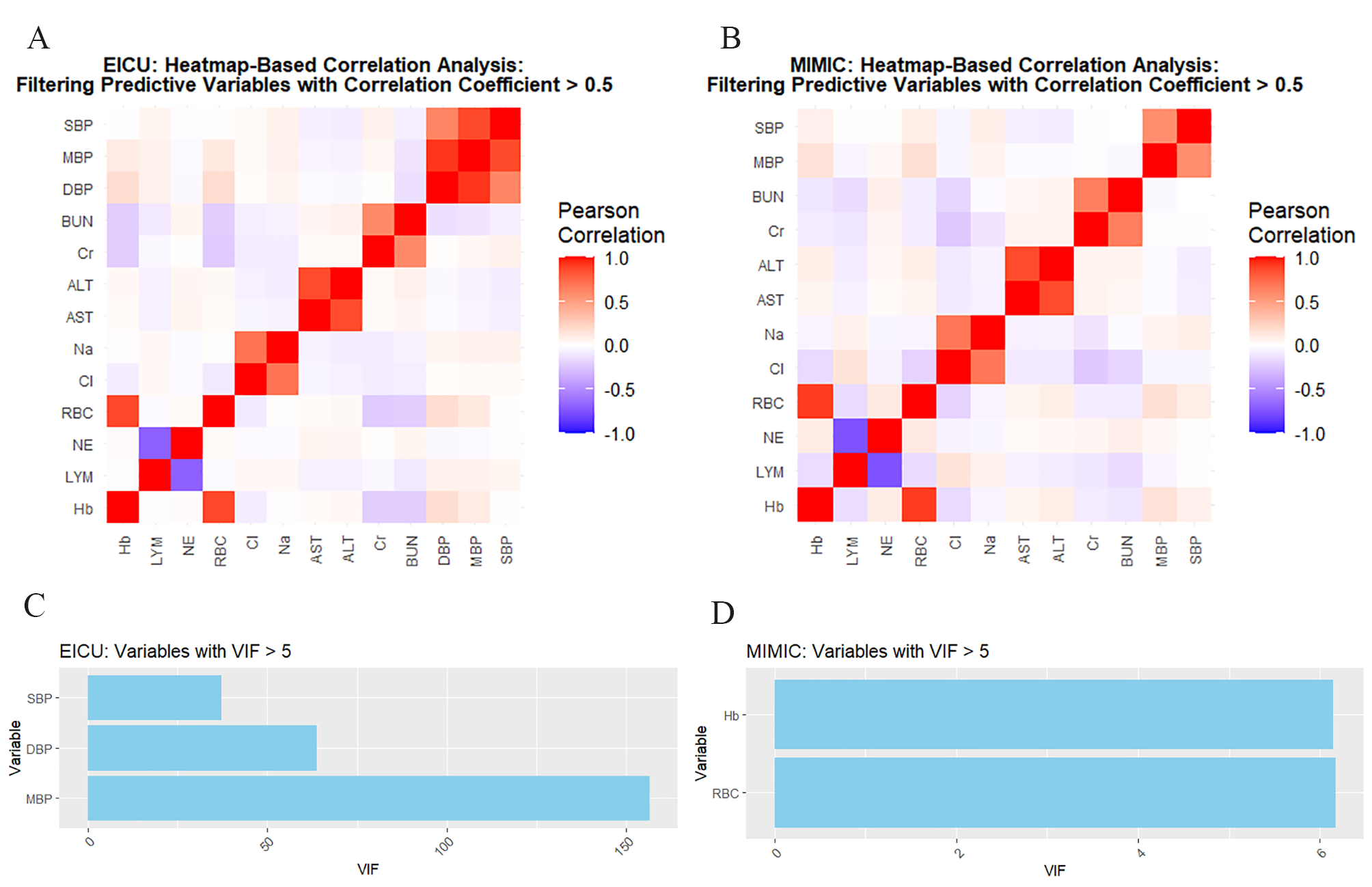

Supplement: Supplementary file 3 [file Image_2.TIF]

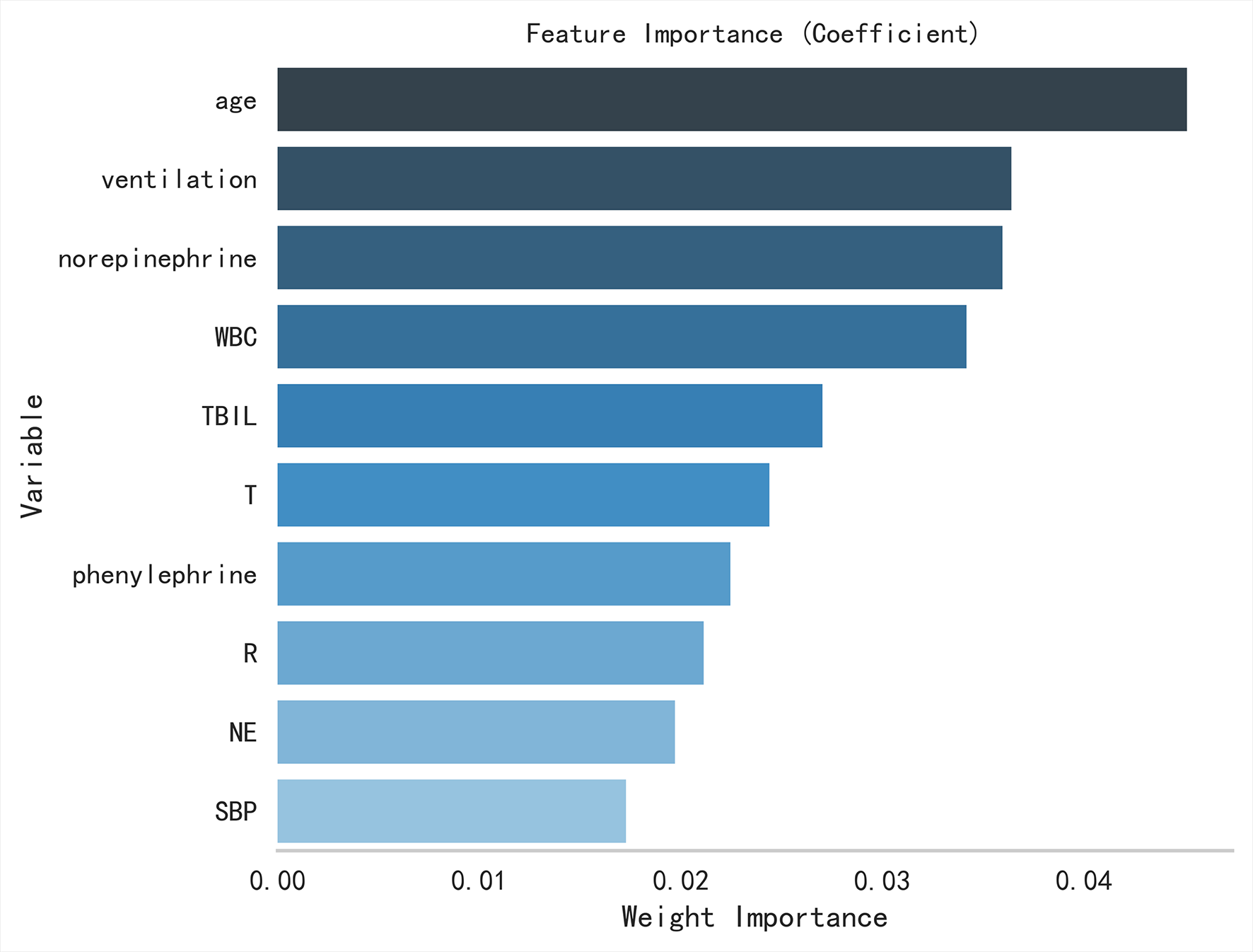

Supplement: Supplementary file 4 [file Image_3.TIF]

**Appendix Table 1: Hyperparameters and Evaluation Metrics for Various Models**
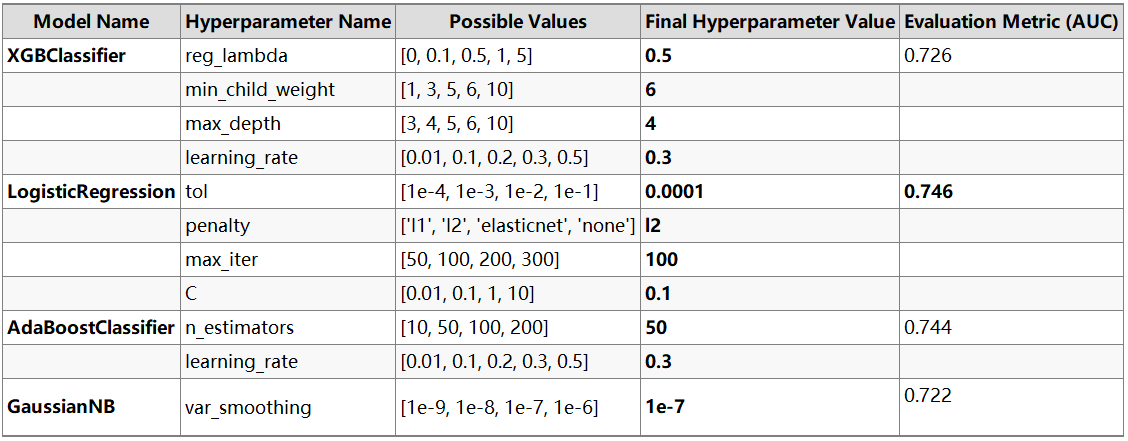

Supplement: Supplementary file 5 [file Table_1.DOCX]
